# Royal Medical and Chirurgical Society

**Published:** 1897-01-16

**Authors:** 


### ROYAL MEDICAL AND CHIRURGICAL SOCIETY.

An important paper was read by Dr. Risien Russell on behalf of himself and Mr. Kingdon, of Nottingham, on a rare and fatal disease of infancy, which they described as "infantile cerebral degeneration, with symmetrical changes at the macula," four cases of which had come under their notice. Although the disease had been already recognised, this had only been done by ophthalmic surgeons, who had dealt mainly with the ocular symptoms. No autopsy had been obtained in any of the recorded cases, except in one reported by Dr. Knapp, in regard to which a description of the changes found in the brain is given by Dr. Sachs, of New York, in a paper entitled, "Arrested Cerebral Development." The authors, however, as the result of their inquiries, looked on the condition as due to degeneration rather than arrest of development. The disease commenced, in the early months of infant life, by muscular enfeeblement, associated with distinctive ophthalmoscopic appearances; complete paralysis resulted later; and the patients died about the end of the second year.

Both male and female children were affected, and no cause could be assigned; but there was a marked racial peculiarity, in that all the children had been Jews. More than one child in the same family was usually affected.

The symptoms and progress of the disease were described as showing three stages. In the first stage a child, born at full term and healthy, began to show signs of muscular enfeeblement and of failure of sight about the end of the third month. About the fourth or fifth month definite and characteristic changes in the region of the macula lutea could be detected on ophthalmoscopic examination.

The second stage of the disease was characterised by such marked muscular enfeeblement that the child was unable to sit up, its head fell backward if unsupported, and its grasp was feeble; when in the recumbent posture, there was inability to turn from side to side. It was generally apathetic, and the face bore an expression of mental enfeeblement. Vision was abolished except that there was still perception of light; but hearing remained acute, and the sense of taste was preserved.

In the third stage the enfeebled muscles became atrophied, and general emaciation progressed and became very marked. The deep reflexes were exaggerated, and still later there was rigidity of the extremities and retraction of the head, while occasional spasmodic contractions caused the child to start and cry from pain. Convulsions were not the rule, but had been noted in two exceptional cases.

The characteristic ophthalmoscopic appearances consisted in symmetrical changes at the macula lutea, in which situation there was a whitish-grey patch, but slightly raised above the surface of the retina, with a few retinal vessels visible on it at the periphery. In the centre of the patch the fovea centralis was seen as a dark cherry-red spot. In the early months the optic papilla showed no decided changes, but later in the course of the disease there was definite optic atrophy.

On post-mortem examination profound changes were met with in the brain and spinal cord. There was widespread degeneration of the pyramidal cells of the cerebral cortex, which was progresssive in character; extensive degeneration of the fibres of the corona radiata and of the pyramidal tracts throughout their whole course in the pons, medulla oblongata and spinal cord. The essential change, however, was in the cortex. All the others were descending degenerations.

The changes in the fundus of the eye seemed to form an essential symptom.

